# HER-SAFE study design: an open-label, randomised controlled trial to investigate the safety of withdrawal of pharmacological treatment for recovered HER2-targeted therapy-related cardiac dysfunction

**DOI:** 10.1136/bmjopen-2024-091917

**Published:** 2025-02-05

**Authors:** Benjamin Dowsing, Hakim-Moulay Dehbi, Robin Chung, Joanna Pedra, Orla Worn, Jessica Artico, Peter Schmid, Rebecca Roylance, Peter Kellman, James C Moon, Tom Crake, Mark Westwood, Arjun Ghosh, Maria Sol Andres, Muhummad Sohaib Nazir, Alexander R Lyon, Daniel Chen, Malcolm Walker, Charlotte H Manisty

**Affiliations:** 1 Institute of Cardiovascular Science, University College London, London, UK; 2 Saint Bartholomew's Hospital Barts Heart Centre, London, UK; 3 Comprehensive Clinical Trials Unit at UCL, London, UK; 4 Whittington Health NHS Trust, London, UK; 5 Imperial College Healthcare NHS Trust Cardiology Service, London, UK; 6 Queen Mary University of London, London, UK; 7 Barts Health NHS Trust, London, UK; 8 Department of Oncology, UCLH Foundation Trust, London, UK; 9 National Heart Lung and Blood Institute, Bethesda, Maryland, USA; 10 Cardio-Oncology Centre of Excellence, Royal Brompton Hospital, London, UK; 11 King's College London School of Biomedical Engineering & Imaging Sciences, London, UK; 12 Cardio-Oncology Centre of Excellence, Royal Brompton and Harefield Hospitals, London, UK

**Keywords:** Heart failure, Breast tumours, Cardiovascular Disease, Cardiovascular imaging

## Abstract

**Introduction:**

A quarter of breast cancers show human epidermal growth factor-2 (HER2) overexpression, where targeted therapy dramatically improves survival. However, cancer therapy-related cardiac dysfunction (CTRCD) occurs in up to 15% of patients. With the interruption of HER2 therapy, if necessary, and the initiation of heart failure therapy (HFT), HER2 CTRCD recovers in over 80% of cases. The need to continue HFT in ‘recovered’ HER2 CTRCD following completion of HER2 therapy is unclear and there are potential significant impacts on patient’s quality of life (QoL). The Randomised Controlled Trial for the Safety of Withdrawal of Pharmacological Treatment for Recovered HER2 Targeted Therapy Related Cardiac Dysfunction (HER-SAFE) aims to evaluate whether HFT can be safely withdrawn in non-high cardiovascular (CV) risk patients with ‘recovered’ HER2 CTRCD.

**Methods and analysis:**

This is a multicentre, open-label randomised controlled trial investigating whether withdrawal of HFT is non-inferior to continuation in non-high CV risk, breast cancer survivors with recovered HER2 CTRCD after cancer treatment completion. The primary endpoint is the incidence of guideline-defined cardiac dysfunction or clinical heart failure. Secondary endpoints include changes in cardiac blood biomarkers, cardiovascular magnetic resonance (CMR)-derived strain and tissue mapping and heart failure symptom questionnaires. The study will recruit 90 participants who will undergo serial clinical assessment over 12 months with advanced cardiovascular imaging (CMR scans with automated analysis at baseline, 6 and 12 months), cardiac biomarker measurement (six time points over 12 months), plus complete heart failure QoL and medication disutility questionnaires. This is the first multicentre study to address this significant clinical issue.

**Ethics and dissemination:**

This study was approved by the research ethics committee (London—London Bridge, 23/LO/0152). The results will be disseminated in peer-reviewed scientific journals.

**Trial registration number:**

NCT05880160.

**STRENGTHS AND LIMITATIONS OF THIS STUDY**

- This is a multicentre study with participant cohort focused on HER2-targeted therapy-related cardiac dysfunction.
- Endpoint assessment using gold standard cardiovascular magnetic resonance (CMR) scans with artificial intelligence analysis for improved measurement precision.
- Unblinded study design, in keeping with other deprescribing trials.
- Cohort restricted to non-high cardiovascular risk patients; therefore, results will not be generalisable to higher risk populations.

## Introduction

### Human epidermal growth factor 2 (HER2+) breast cancer and HER2-targeted therapies

Breast cancer is the most common cancer worldwide, affecting more than 2 million people annually.^1^ More than 75% of patients now survive for 10 years or longer, with better life expectancy with early-stage disease.^2^ Overall, 15–20% of breast cancers overexpress the human epidermal growth factor 2 (HER2) gene and historically had poorer outcomes.^3 4^ HER2-targeted therapies dramatically improve survival,^5^,^7^ but are associated with acute, reversible cardiotoxicity.^8^,^10^ The overall reported incidence of symptomatic heart failure is 1.9%, while asymptomatic declines in left ventricular ejection fraction (LVEF) occur more commonly (approximately 15%), with higher rates when coadministered with anthracycline chemotherapy.^8 11 12^ This cardiotoxicity can lead to premature interruption or discontinuation of HER2 therapies, which is associated with worse cancer outcomes.^13 14^

### HER2-targeted therapy-related cardiac dysfunction

Unlike other cancer therapy-related cardiac dysfunction (CTRCD), HER2-targeted therapy-related cardiotoxicity is generally reversible on treatment discontinuation,^15^,^17^ and late cardiotoxicity is uncommon.^15 18^ For patients with early breast cancer, the duration of HER2-targeted therapy is 12 months and normalisation of cardiac function occurs in most patients; over 80% of patients with breast cancer and HER2 CTRCD show recovery at a median of 7 months post initial dysfunction.^15^ Myocyte apoptosis/necrosis is rare, and myocyte dysfunction is thought to be due to blockade of cardioprotective HER2 signalling and increased oxidative stress.^19 20^ The European Society of Cardiology (ESC) 2022 cardio-oncology guidelines recommend HER2-targeted therapies should be reviewed (with consideration to interrupting) for LVEF declines of >10% from baseline to <50%, with prompt initiation of heart failure treatments (HFTs), consisting of an ACE inhibitor (ACEi)/angiotensin receptor blocker (ARB) and/or a beta-blocker.^21^

There is, however, no evidence to guide the duration of this HFT in patients who have completed HER2-targeted therapy and whose cardiac function has ‘recovered’ (that is LVEF returned to >50%, the absence of cardiac symptoms and N-terminal pro-B-type natriuretic peptide (NTpro-BNP) in the normal range). The TRED-HF (Withdrawal of pharmacological treatment for heart failure in patients with recovered dilated cardiomyopath) study^22^ demonstrated that relapse was common in patients with recovered dilated cardiomyopathy (DCM) following HFT withdrawal, though the cohort and pathophysiology differ greatly, and the results may not be generalisable. As such, international society guidelines do not address the common clinical question of HFT continuation in recovered HER2 CTRCD.

### Evidence for medication withdrawal in recovered heart failure

Across general heart failure aetiologies, international guidelines recommend continuation of HFT indefinitely, even when cardiac function and natriuretic peptides normalise, to prevent relapse in cardiac dysfunction.^22 23^ Results from the TRED-HF study^22^ found a relapse rate of 44% over 6 months in patients with (predominantly idiopathic) DCM who underwent complete withdrawal of HFT, with zero relapse events in those continuing treatment. Cardiovascular magnetic resonance (CMR) imaging analysis from this study demonstrated that withdrawing therapy caused rapid adverse remodelling, with early tissue and functional changes, even among patients who did not relapse—notably an increase in left ventricular (LV) mass, cell mass, matrix mass and a reduction in global longitudinal strain (GLS).^24^ Other data have similarly found evidence of relapse on withdrawal of spironolactone in recovered DCM.^21^ This supports long-term prescription of HFT in patients with DCM, even for asymptomatic patients with normalisation of LVEF and biomarkers.

Patients in TRED-HF, however, differ from those with HER2 CTRCD, for whom LV impairment is generally reversible and where the cause is removed when cancer treatment completes. The risks of an ongoing pathophysiological process were high in TRED-HF with scar on CMR in 39% and truncating titin (TTN) gene mutations in 22%. Participants in TRED-HF had recovered from severe clinical heart failure (mean diagnosis LVEF 25%), atrial fibrillation in 24% and heart failure hospitalisation in 63%; whereas HER2 CTRCD is often detected during routine surveillance screening, in younger asymptomatic patients without pre-existing cardiovascular (CV) disease and with mild reductions in LVEF.^25^ High scar burden on CMR, higher B‐type natriuretic peptide level and lower LVEF at the initial LVEF recovery have been demonstrated as independent predictors of a relapse in cardiac dysfunction in patients with DCM^26^; factors that are again less common in patients with breast cancer and HER2 CTRCD.

### Medication disutility

An estimated 30 000 women under the age of 45 years will be diagnosed with breast cancer in the USA in 2024 and there are over 4 million living breast cancer survivors.^27^ Long-term, potentially life-long, compliance with HFT is challenging and can have significant impact on quality of life (QoL), especially in a younger cohort of patients where this may impact family planning.^28^ HFT is therefore commonly continued for at least 6 months post recovery, with subsequent individualised decision-making regarding stopping. This includes an assessment of concomitant comorbidities, CV risk factors, patient preference, level of physical activity and medication tolerance.^29^ This is, however, not evidence based, with no formal consensus recommendations to aid clinicians, hence practice varies widely.

Medication disutility (the inconvenience of taking a medication) is generally higher than recognised by clinicians—particularly in the preventative setting.^30^ It has been assessed formally for statins and bisphosphonates where patients on these medications would trade 4 or 6 months, respectively, of good health over 10 years to avoid taking them.^31^ The disutility of HFT has not been assessed. Formal exploration of patients’ perceptions of medication disutility in the setting of ‘recovered’ CTRCD has also not been performed, which implies that the impact of drug side effects and lifestyle choices on patient motivations are unclear.

## Methods and analysis

### Study design

**Figure 1:**
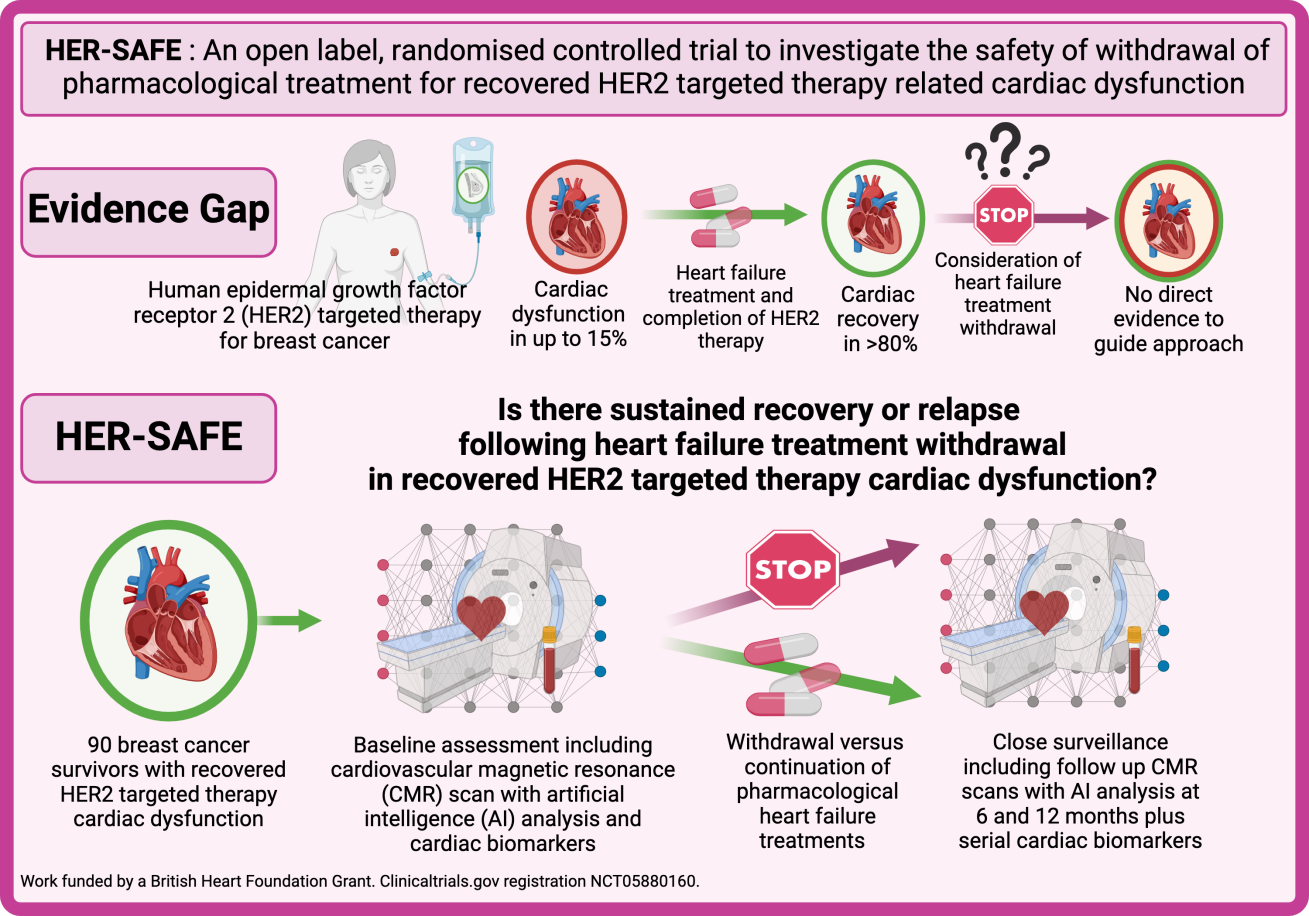
Central illustration. Randomised Controlled Trial for the Safety of Withdrawal of Pharmacological Treatment for Recovered HER2 Targeted Therapy Related Cardiac Dysfunction (HER-SAFE). There is an evidence gap in the management of breast cancer survivors with recovered HER2-targeted therapy cardiac dysfunction that this study will address.

This is a UK multicentre, open-label, randomised controlled trial to compare the phased withdrawal versus continuation of HFT for ‘recovered’ HER2 therapy-related cardiac dysfunction in patients with treated breast cancer at non-high CV risk.(figure 1).

**Figure 2:**
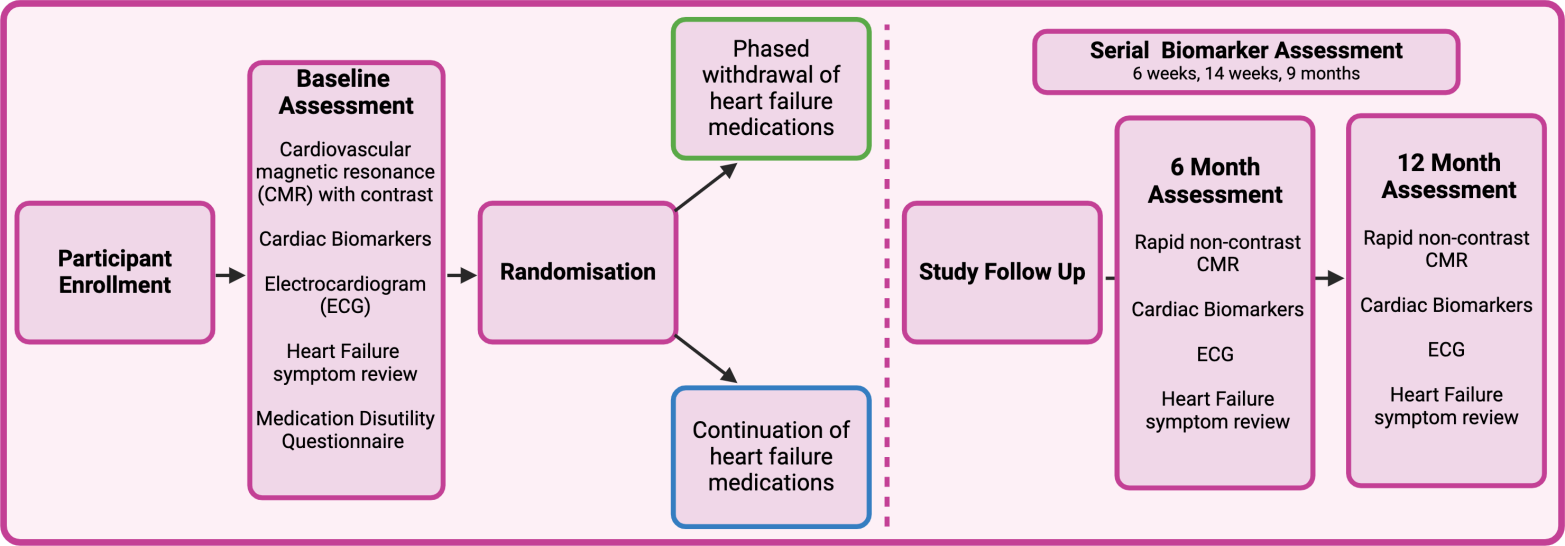
Schematic of the Randomised Controlled Trial for the Safety of Withdrawal of Pharmacological Treatment for Recovered HER2 Targeted Therapy Related Cardiac Dysfunction (HER-SAFE). Following enrolment and baseline assessment, participants are randomised to withdrawal or continuation of their heart failure treatment and then followed up for 12 months.

‘Recovery’ is defined as the absence of heart failure symptoms, with LVEF>50% for at least 6 months and NTpro-BNP<125 ng/L. CV risk is defined according to their cardiotoxicity risk status prior to HER2 therapy, as per the specific HER2-targeted therapies risk calculator from the Heart Failure Association and International Cardio-Oncology Society (HFA-ICOS) 2020 position paper on baseline cardiotoxicity risk assessment in patients with cancer; this is a risk score based on expert opinion, where the risk of future cardiotoxicity was considered as: low risk<2%, medium risk 2–9%, high risk 10–19% and very high risk≥20%.^25^ Table 1 describes the study as per the Consolidated Standards of Reporting Trials guidelines,^32^ and figure 2 illustrates the trial design.

**Table 1:** ‘HER-SAFE’ extended Consolidated Standards of Reporting Trials guidelines items

	Description
Title	HER-SAFE: an open-label, randomised controlled trial to investigate the safety of withdrawal of pharmacological treatment for recovered human epidermal growth factor 2 (HER2)-targeted therapy-related cardiac dysfunction.
Trial design	Open-label, non-inferiority, randomised controlled trial.
Inclusion	Adult breast cancer with low or medium risk by baseline Heart Failure Association and International Cardio-Oncology Society (ICOS) cardiotoxicity assessment,^25^ who have completed HER2-targeted therapy, demonstrate recovered HER2-targeted therapy-related cardiac dysfunction (left ventricular ejection fraction>50% for 6 months, no heart failure symptoms and N-terminal pro-B-type natriuretic peptide and continued heart failure treatment since then (at least one of a beta-blocker and/or ACE inhibitor/angiotensin receptor blocker).
Exclusion	Ongoing cancer treatment for metastatic disease, life expectancy less than 12 months, anthracycline therapy-related cardiac dysfunction, any other known cardiomyopathy or the presence of another guideline-recommended indication to continue their heart failure medications.
Intervention	The phased withdrawal of pharmacological heart failure treatments.
Objective	To evaluate whether phased withdrawal of pharmacological heart failure therapy in patients with non-high cardiovascular risk breast cancer and ‘recovered’ HER2-targeted therapy-related cardiac dysfunction, after completion of cancer therapy, is non-inferior to continuation with regard to risk of recurrence in cardiac dysfunction.
Outcome	The incidence of relapsed cardiac dysfunction over 12 months, based on the ICOS 2021 guidelines^27^ definitions of cancer therapy-related cardiac dysfunction (CTRCD).
Randomisation	Minimisation using a biased coin probability method (70%) and marginal balance distance method. Minimisation factors are: current age over 65 years, baseline cardiovascular level greater than low,^4^ CTRCD severity greater than mild,^27^ cumulative anthracycline dose greater than 250 mg/m^2^ and any radiotherapy to the left breast.
Blinding	Unblinded.
Recruitment	Target: 90 participants. Power: 74 participants.
Recruitment	Open.
Registration	Clinicaltrials.gov: NCT05880160.London Bridge Research Ethics Committee (REF: 23/LO/0152).
Funding	British Heart Foundation (FS/CRTF/22/24395).

HER-SAFERandomised Controlled Trial for the Safety of Withdrawal of Pharmacological Treatment for Recovered HER2 Targeted Therapy Related Cardiac Dysfunction

### Study objectives

The primary objective of the study is to determine whether phased withdrawal of pharmacological HFT in patients with breast cancer and ‘recovered’ HER2-targeted therapy-related cardiac dysfunction, after completion of cancer therapy, is non-inferior to continuation with regard to risk of recurrence in cardiac dysfunction.

The secondary objectives of the study are to assess:

1. If relapse in cardiac dysfunction occurs, whether baseline CMR and biomarkers prior to treatment withdrawal predict subsequent relapse.
2. How patient ratings of medication disutility depend on age, comorbidities, medication side effects and perceptions of disease severity.

### Participant selection

Participants who meet the required inclusion and exclusion criteria as shown in table 2 are eligible for enrolment into the study. Eligible participants will have completed HER2-targeted therapy as part of breast cancer treatment. Participants will be low or medium risk by baseline HFA-ICOS cardiotoxicity assessment.^25^ Participants will have been previously diagnosed and treated for HER2 CTRCD (meeting ESC guideline CTRCD definitions^33^), continued HFT since then (at least one of a beta-blocker and/or ACEi/ARB) and subsequently ‘recovered’. ‘Recovery’ of cardiac function is based on the prior definition^22^ of an LVEF of greater than 50% for 6 months (by echocardiography or CMR) and an NTpro-BNP within normal limits (125 ng/L).

**Table 2:** Inclusion and exclusion criteria for ‘HER-SAFE’

Participant inclusion criteria	Participant exclusion criteria
Age>18 years old.	Any ongoing indications for HER2-targeted therapy.
Human epidermal growth factor 2 (HER2) positive, non-metastatic breast cancer.	Life expectancy<12 months.
Completion of HER2-targeted therapy.	High/very high baseline cardiovascular risk according to the Heart Failure Association and International Cardio-Oncology Society risk stratification.^25^
A prior diagnosis of HER2-targeted therapy-related cardiac dysfunction.	LVEF<50% prior to HER2-targeted therapy initiation or on completion of anthracycline treatment.
Cardiac function has ‘recovered’. ‘Recovery’ is defined as absence of heart failure symptoms with left ventricular ejection fraction (LVEF) increased to >50% for 6 months and N-terminal pro-B-type natriuretic peptide<125 ng/L.	Other ongoing guideline recommended indications for ACE inhibitors/angiotensin receptor blockers and/or beta-blockers.
Currently taking heart failure medications (at least any ACE inhibitor/angiotensin receptor blockers and/or beta-blocker).	Absolute contraindications to cardiovascular magnetic resonance.

HER-SAFERandomised Controlled Trial for the Safety of Withdrawal of Pharmacological Treatment for Recovered HER2 Targeted Therapy Related Cardiac Dysfunction

Exclusion criteria include ongoing cancer treatment for metastatic disease, life expectancy less than 12 months or another guideline-recommended indication to continue their specific HFT (including type 2 diabetes mellitus with hypertension who would be recommended to continue an ACE inhibitor/ARB regardless).^34^ Those with anthracycline CTRCD or other cardiomyopathies are also excluded—though patients may have received anthracyclines in their cancer treatment.

Participants will be recruited from local secondary care cardio-oncology and breast cancer services. Following approach by the study team potential participants will receive a copy of the participant information sheet and written informed consent (online supplemental material) will be obtained prior to any study visits.

### Randomisation procedure

Eligible participants will be randomised by a minimisation technique in favour of the arm that minimises imbalance between groups. This is conducted via dedicated peer reviewed open-source software,^35^ using a biased coin probability method (70%) and marginal balance distance method. Minimisation factors are: current age over 65 years, HFA-ICOS baseline cardiotoxicity risk stratification for HER2-targeted cancer therapies greater than low,^25^ CTRCD severity greater than mild,^36^ cumulative anthracycline dose greater than 250 mg/m^2^ and any radiotherapy to the left breast. This represents factors identifying those at the highest future CV risk at end of cancer treatment assessment.^33^

### Study schedule

The study schedule (online supplemental table 1) summarises procedures to be performed at each visit. Participants will undergo baseline assessment, randomisation to the controlled or experimental group and then follow-up over 12 months. At baseline, participants will undergo a comprehensive CMR with parametric mapping (T1, T2, extracellular volume) and contrast administration for early and late gadolinium enhancement imaging, to exclude patients with other causes of the prior cardiomyopathy including prior infarction.

Patients will undergo clinical assessment, ECG, cardiac biomarker testing (NTPro-BNP and high-sensitivity troponin; Roche platform electrochemiluminescence assays) and will complete heart failure QoL questionnaires (Kansas City Cardiomyopathy and Minnesota Living with Heart Failure), plus a medication disutility questionnaire (online supplemental material).

Follow-up visits will be at 6 and 12 months and participants will undergo a ‘rapid’ non-contrast CMR (including T1 and T2 parametric mapping), ECG, physical assessment, cardiac biomarker testing and complete heart failure QoL questionnaires.

At 6 weeks, 14 weeks and 9 months, participants will also have clinical assessment including symptoms, vital signs and cardiac biomarker testing.

Participants will be offered longer term follow-up outside the study period with subsequent data collection for 5 years, via annual clinical surveillance from the study sites cardio-oncology services.

### Phased medication withdrawal

**Figure 3:**
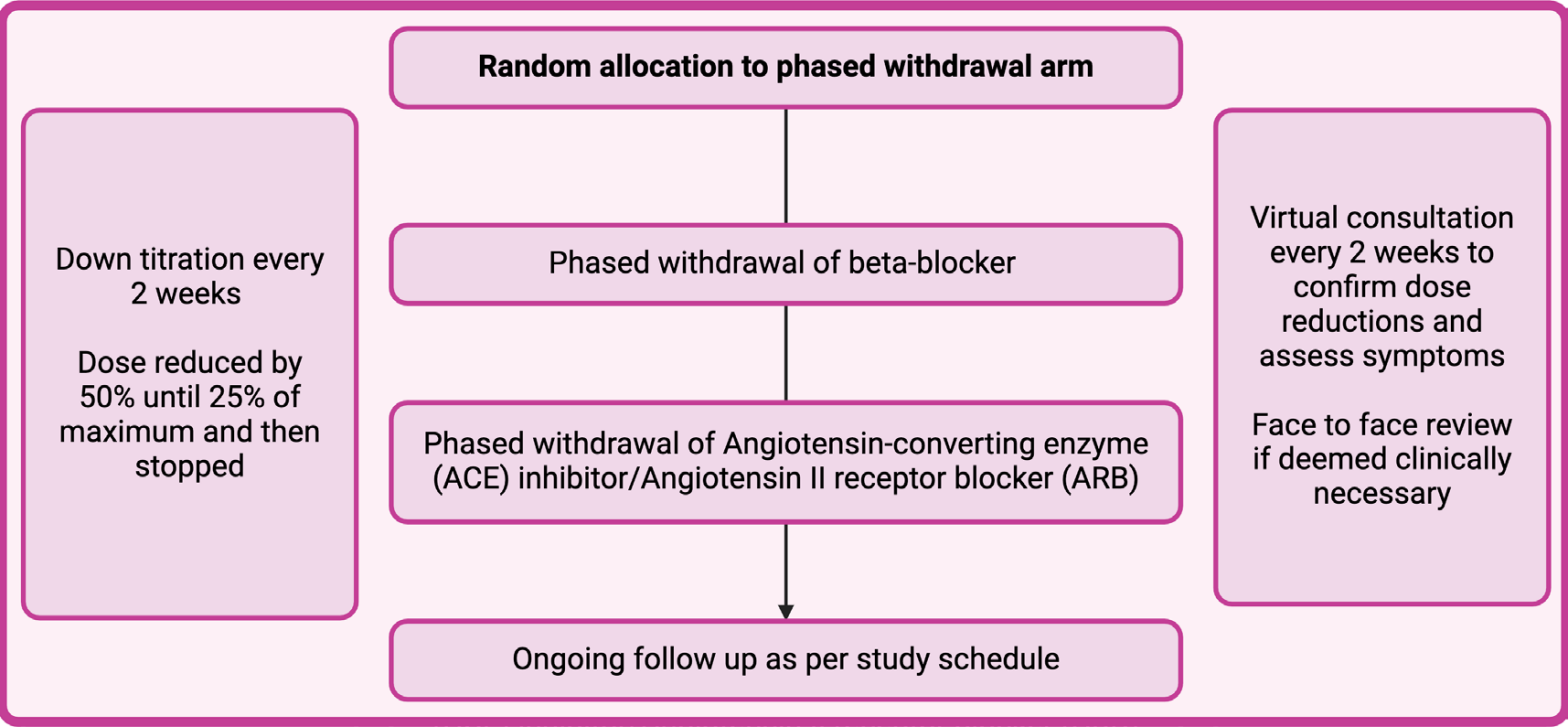
Phased withdrawal of heart failure medications—medications are stopped sequentially and, in a step, wise approach.

Participants in the intervention arm will undergo phased withdrawal of heart failure medications according to a prespecified algorithm based on published algorithms.^22^ This had been designed following extensive consultation with independent experts and attempts to mimic ‘real-world’ medication withdrawal in clinical practice. Medications will be down titrated in a phased process every 2 weeks over a maximum of 16 weeks. Drug doses will be reduced by 50% in a stepwise manner every 2 weeks, until the patient is taking 25% or less of the maximum recommended dose at which point the medication will be stopped—before moving on to the next medication (figure 3). The controlled group will remain on their current HFTs.

### Sample size calculation

TRED-HF^22^ recruited 51 participants with 25 randomised to treatment withdrawal, 26 to treatment continuation. ‘Relapse’ outcome events occurred in 11/25 withdrawal patients within 6 months, with 0/25 outcomes in the continuation participants. Our retrospective pilot data (using local cardio-oncology service records) demonstrated 0/35 and 0/12 ‘relapse’ outcomes in study eligible patients who continued HFT compared with those who withdrew respectively (median 6 months of follow-up, IQR 6–10). Following discussion with patients from our pilot dataset, a 10% relapse rate following HFT withdrawal was considered acceptable, provided participants are under close surveillance. Using a binary outcome (‘relapse’ vs ‘no relapse’) non-inferiority design with α=0.05 (one sided) and 80% power, 74 participants (37 per study arm) are required to exclude a difference in favour of the controlled group of more than 10 percentage points. Assuming a 10% dropout rate, a sample size of n=90 is therefore considered sufficient.

### Cardiac imaging protocols and analysis

**Figure 4:**
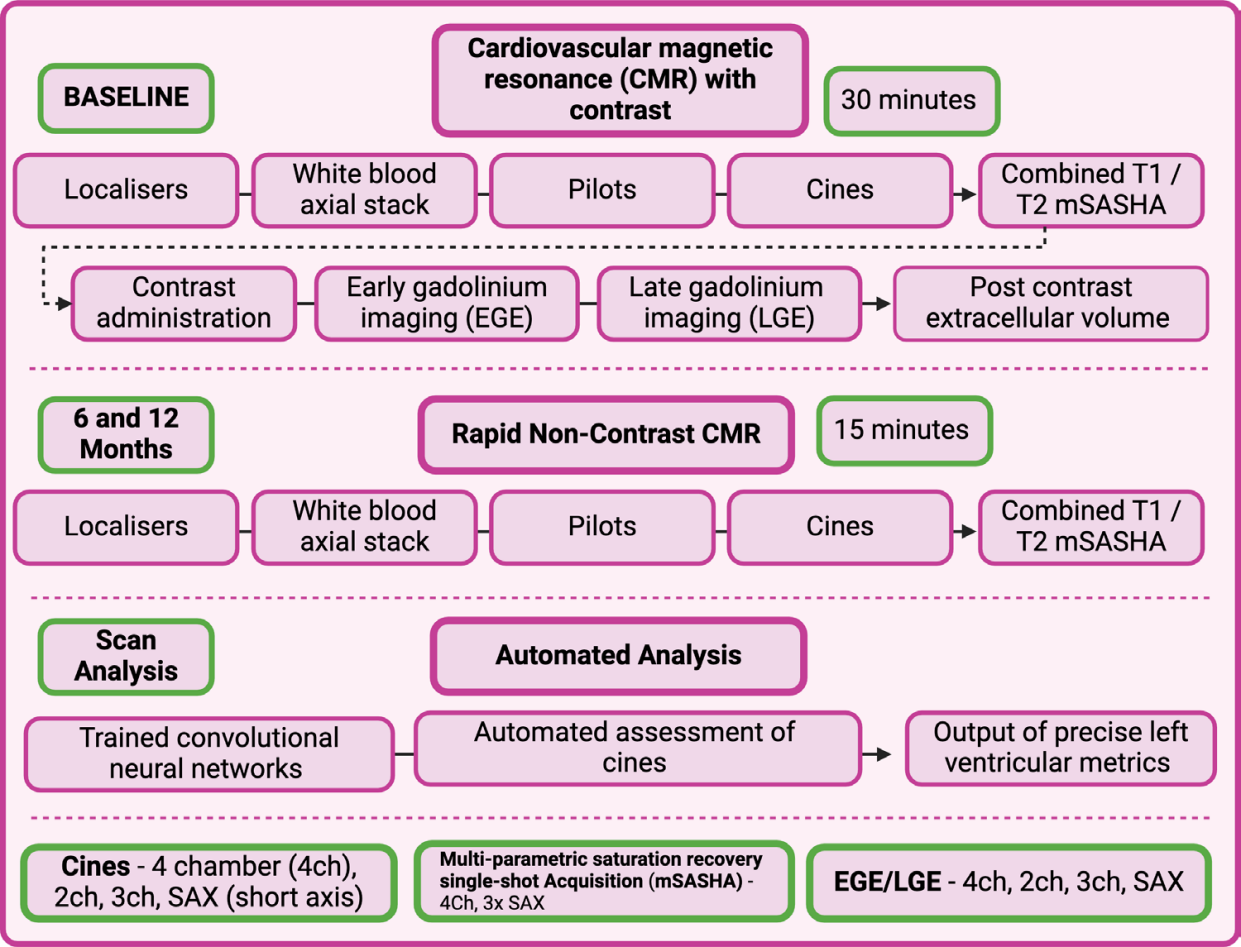
CMR schematic. Participants undergo a baseline CMR with contrast and two further ‘rapid’ CMR at 6-month and 12-month follow-up, respectively. The protocols are described here.

Participants will undergo a CMR study (1.5 T) with gadolinium-based contrast administration at baseline and ‘rapid’ non-contrast CMR study at 6 and 12 months (figure 4 and online supplemental tables 2 and 3). Parametric tissue mapping (T1 pre and post contrast, and T2) will be performed. Analysis of cardiac volumes, ejection fraction and GLS will be performed in a central CMR Core Lab with analysts blinded to treatment arm. A fully automated machine learning algorithm, based on trained convolutional neural networks, will segment the LV blood volume and myocardium, before outputting precise measurements of LV structure and function.^37^

### Medication disutility

Medication disutility will be assessed by a structured interview using an adapted questionnaire (online supplemental material), based on prior published work on real-world medication disutility.^30^ Specifically, this will assess the gain in healthy CV lifespan required by each participant to offset the disutility of continuing their heart failure medications.

### Endpoints

The primary endpoint of the study is the incidence of recurrent CTRCD over the course of 12 months following randomisation, as defined by the ICOS 2021 consensus statement^36^ as (at least one of):

- Asymptomatic LVEF reduction by ≥10 percentage points to an LVEF of <50%.
- Asymptomatic LVEF reduction by ≥5 percentage points to an LVEF of <50% plus new relative decline in GLS by >15% from baseline AND/OR new rise in cardiac biomarkers (greater than twofold increase in NTpro-BNP to >400 ng/L, or high-sensitivity troponin>99th percentile).
- Clinical heart failure (based on symptoms and clinical examination) with at least one of the following: fall in LVEF≥5%, increase in cardiac biomarkers (as above), relative fall of more than 15% in GLS, new arrhythmia (excluding ectopy).

The secondary endpoints are change in cardiac biomarkers (high-sensitivity troponin and NTpro-BNP), heart failure symptom scores (Kansas City Cardiomyopathy Questionnaire and Minnesota Living with Heart Failure Questionnaire) with exploratory outcomes assessing CMR-derived LV volumes, ejection fraction, strain and myocardial T1 at 6 and 12 months compared with baseline. Other clinical endpoints will also be reviewed (arrhythmia, acute coronary syndromes, reinitiation of ACEi/ARB or beta-blocker for other clinical indications).

### Blood storage

A plasma and serum sample will be collected for frozen storage at baseline, 6 months and 12 months. Participants will additionally be consented for DNA analysis for genes associated with cardiomyopathy (in the event of a relapse) and fully anonymised data sharing with external academic organisations—for the purpose of research in this area.

### Data analysis plan

For the primary endpoint, non-inferiority will be examined using the CI for the absolute difference in heart failure relapse events (based on the ESC guidelines CTRCD definitions) between groups. Prespecified cardiac events (relapsed CTRCD) will be tabulated by type (symptomatic vs asymptomatic) and severity (mild vs moderate vs severe); and summarised as cumulative frequencies over time. Survival analysis will be summarised as Kaplan-Meier curves and log-rank test. Participants will be analysed according to the intention-to-treat principle. The Randomised Controlled Trial for the Safety of Withdrawal of Pharmacological Treatment for Recovered HER2 Targeted Therapy Related Cardiac Dysfunction (HER-SAFE) is not powered to address changes in the noted secondary outcomes. These analyses will be hypothesis generating.

The medication disutility survey data will be summarised using simple measures of central tendency (mean and median) and spread, further analysed across groups for age groups, level of comorbidities, degree of medication side effects and perceptions of disease severity. The distribution of medication disutility will be examined to assess whether it follows a normal distribution and whether it had the same shape in each group. Differences on tablet disutility across groups will be tested using parametric and non-parametric tests for both.

### Patient and public involvement (PPI)

The study design was discussed with patient groups and presented at a PPI event. Results of the study will be disseminated to the study participants on request and presented at further local PPI events.

### Ethics and dissemination

The investigator and sponsor of the study will adhere to all the relevant guidance, laws and statutes applicable to the performance of clinical trials. The trial will be conducted in accordance with the terms and conditions of the favourable ethical approval given to the trial by the London Bridge Research Ethics Committee (REF: 23/LO/0152) and Health Research Authority in the UK. Findings from the study will be published in peer-reviewed journals and disseminated at national and international conferences. Data will be stored in a secure manner and the study is registered in accordance with the UK Data Protection Act 2018 and General Data Protection Regulation. The study is registered on clinicaltrials.gov (NCT05880160).

## Discussion

We present the study design for ‘HER-SAFE’, the first randomised controlled trial of HFT withdrawal in patients with recovered CTRCD—specifically breast cancer survivors who experienced HER2-targeted therapy cardiotoxicity. This is a multicentre study with endpoint assessment using gold standard CMR with artificial intelligence (AI) analysis, for improved measurement precision and incorporation of a novel medical disutility questionnaire.

HER-SAFE could enable breast cancer survivors to discontinue cardiac medications without the fear of early relapse; improving QoL and reducing the healthcare resources required for lifelong CV follow-up. If participants do relapse, the results from this study may identify predictors that will identify those who will benefit most from remaining on HFT. For clinicians, this will provide, for the first time, an evidence base of how to manage this area of profound clinical uncertainty.

### HFT withdrawal—recovery versus remission

The ongoing management of heart failure patients with normalised cardiac function is a growing area of interest, and it remains uncertain whether this improvement should be labelled as true recovery or HFT assisted remission. Given the significant proportion of relapse seen in the DCM-focused TRED-HF study, on HFT withdrawal, the term heart failure with reduced ejection fraction in remission (HFrEFrem) may be used in this cohort.^38^ The future TRED-HF 2 study (NCT06091475)^39^ will explore the withdrawal of mineralocorticoid receptor antagonists and/or sodium–glucose cotransporter 2 inhibitors, while ACE inhibitors and beta-blockers continue, in DCM patients with HFrEFrem. The ongoing Maintenance of recovered dilated cardiomyopathy patients with half-dose neurohumoral blockades (MED-CHARM) study (ChiCTR2100054051)^40^ is comparing halving the dose of HFT to continuation in DCM patients with HFrEFrem.

HER2 CTRCD with its objective relationship to an apparent precipitant appears promising as an area where ‘true recovery’ may occur. In keeping with our pilot data, a recent retrospective matched cohort study demonstrated stable cardiac function, after a median 29 months, following HFT withdrawal in 10 patients with breast cancer and recovered anthracycline and/or trastuzumab-related cardiac dysfunction.^41^ Though genetic variants associated with DCM have been identified in patients with acute myocarditis^42^ and alcohol induced cardiomyopathy,^43^ conditions which could also be framed as a single triggering precipitant, and unrecognised variants in cardiomyopathy associated genes (most commonly TTN-truncating variants) are also associated with a higher risk of anthracycline CTRCD.^44^ This emphasises that CTRCD may be related to ‘multiple hit’ pathophysiology—leading to ongoing uncertainty regarding sustained recovery of cardiac function following HFT withdrawal and highlighting the need for evidence specific to these groups.

HER-SAFE is planned as a pilot study of early outcomes; larger long-term studies will be needed for definitive confirmation of the safety of HFT withdrawal. The Multi-Centre Non-Inferiority Randomized Controlled Trial of STOPping Cardiac MEDications in Patients With Normalized Cancer Therapy Related Cardiac Dysfunction (STOP-MED CTRCD) (NCT06183437),^45^ due to commence in later 2024, will explore medication withdrawal in a cohort of cancer survivors with prior anthracycline and/or HER2-targeted therapy-related cardiac dysfunction in a larger group (recruitment target 335 participants) with 5 years of planned follow-up.

### Precise cardiac surveillance using CMR with AI analysis

CMR has higher precision (test–retest reproducibility) than standard of care echocardiography for serial screening.^46 47^ However, conventional CMR protocols are long and require time-consuming manual analysis, thereby limiting widespread adoption, particularly for cardiotoxicity surveillance. Recent advances have validated the feasibility of deploying rapid CMR abbreviated protocols,^48^ alongside developing automated analysis using machine learning algorithms to improve CMR speed and precision.^37 49^ This is further supported by AI measures of myocardial contractility using global longitudinal shortening^50^; recommended for early cardiotoxicity detection in cardiotoxicity screening.^51^ Using rapid CMR protocols with integrated AI analysis allows for highly precise measurement of cardiac function, thus identifying any true dysfunction at an early stage. This high precision also allows for a smaller sample size when using LVEF endpoints—with a potential 46% reduction compared with human analysis.^37^

### Breast cancer survivorship and medication disutility

Breast cancer survivors already face ongoing medical and psychosocial challenges. ‘Am I healthy or am I not?’ has been identified as a key theme in survivorship and a significant source of health anxiety.^52^ Breast cancer survivors express a strong desire for a ‘return to normalcy’—be that work, exercise or family life.^53^ QoL and prevention of treatment-related complications have also been highlighted as priorities by cancer survivors.^54^ Further labels of a chronic illness, like heart failure, prevent this and have a significant effect on breast cancer survivors’ QoL.^28^ Breast cancer survivors with recovered HER2 CTRCD are currently treated as though they have a lifelong chronic disease, despite recovery of cardiac function and removal of the precipitant (HER-2-targeted therapies).

The use of lifelong medications (even those perceived as common or safe) also has significant costs not only to healthcare systems,^55^ but the individual.^56^ These costs, be they financial, physical or psychological, are often unseen and overlooked by healthcare providers.^57^ Taking daily medications is widely perceived by healthcare professionals to have minimal burden, but qualitative evidence from patients with chronic conditions suggests this is not the case—‘‘You say treatment, I say hard work’.^57^ Observed medication disutility has been measured to range from 1 day to>10 years of life being required by subjects to make daily preventative therapy worthwhile.^30^ Meanwhile, up to 40% of adults in a large, national sample would decline a daily pill to prevent CV disease—even if this shortened their life.^56^ The rationale for continuation of a ‘secondary preventative’ medication when the precipitant has gone, and where clear long-term benefit is unproven, cannot justify the inconvenience of long-term combination medication use.^58^ Alongside this, the healthcare resource implications associated with follow-up and administration of HFT indefinitely are significant.^55^ We will assess this ‘medication disutility’ for the first time in both breast cancer survivors and CTRCD.

### Trial status

The protocol version number and date：V.6.0, 31 July 2024. The study was conceived and designed in 2022. Enrolment began in 2023 and is expected to end in March 2025. At the time of manuscript preparation, more than 71 participants had been enrolled. Enrolment in this study was ongoing at the time of manuscript submission.

## supplementary material

10.1136/bmjopen-2024-091917 online supplemental file 1
