# Supplementary material for: HER-SAFE study design: an open-label, randomised controlled trial to investigate the safety of withdrawal of pharmacological treatment for recovered HER2-targeted therapy-related cardiac dysfunction
Source: BMJ Open. 2025 Feb 5;15(2):e091917. doi: 10.1136/bmjopen-2024-091917 (PMC11800297; doi:10.1136/bmjopen-2024-091917)
Supplement: online supplemental file 1 [file bmjopen-15-2-s001.docx]

# Supplemental Material - HER-SAFE Study Design Paper

## Table 1. Study Schedule.

| **Assessment** | **Baseline** | **6 Weeks** | **14 Weeks** | **6 Months** | **9 Months** | **12 Months** |
| --- | --- | --- | --- | --- | --- | --- |
| Medical History | X |  |  |  |  |  |
| Symptom Assessment | X | X | X | X | X | X |
| Physical Examination | X |  |  | X |  | X |
| Vital Signs (BP, HR) | X | X | X | X | X | X |
| ECG | X |  |  | X |  | X |
| Cardiac MRI with Contrast | X |  |  |  |  |  |
| Rapid Non-contrast MRI |  |  |  | X |  | X |
| Cardiac Biomarkers | X | X | X | X | X | X |
| Haematology | X |  |  | X |  | X |
| Biochemistry | X |  |  | X |  | X |
| Serum and Plasma Storage | X |  |  | X |  | X |
| Heart Failure Symptom Questionnaires | X |  |  | X |  | X |
| Medication Disutility  Questionnaire | X |  |  |  |  |  |

## Table 2. Cardiovascular Magnetic Resonance (CMR) with contrast - Baseline - 1.5 Tesla (T)

| Cardiovascular Magnetic Resonance (CMR)Technique | CMR Sequence | Scan Prescription | Typical Scan Parameters |
| --- | --- | --- | --- |
| Localizer and Pilots | - Bright blood single shotfast spin-echo stack. - Steadystate free precession (SSFP) | - 3 orthogonal slices (axial, coronal, sagittal) - Transverse stack covering the heart   Standard cardiac planes:   - 2-chamber - 4-chamber - 3-chamber - Short axis stack (5 slices) | - Repeat time (TR): 2.37ms - Echo time (TE): 1.2ms - Slice thickness 8mm - Field of view (FOV): 360 x 360mm - Red matrix 256 - Flip angle 60. |
| Cine Imaging | - SSFP | Standard cardiac planes:   - 2-chamber - 4-chamber - 3-chamber - LVOT - Aortic valve - Short axis stack | - Retrospective ECG gating - TR: 2.7ms - TE: 1.1ms, - Flip angle 80° - Grappa factor 2 - voxel size 1.8x1.8mmx6mm - x-y spatial resolution1 to 2mm/pixel - slice thickness 8mm.   For SAX:   - inter-slice gap 2mm - 10 segments (true temporal resolution 27ms, reconstructed temporal resolution 22-33) |
| Tissue Mapping | - multiparametric SAturation‐recovery single‐SHot Acquisition (mSASHA) - pre- Modified Look-Locker inversion recovery (MOLLI)* - post-MOLLI*   *(extracellular volume [ECV] generation) | - mSASHA: 4Ch, SAX (3 slices) - pre/postMOLLI: 4ch, SAX (1 mid slice) | - TR: 9.0ms - TE: 1.3ms - FOV 360 x 360 mm - Slice Thickness: 8mm |
| Contrast Administration | Gadolinium based contrast injection by hand (dose 0.1mmol/kg) | | |
| Early Gadolinium Enhancement | - SSFP | Standard cardiac planes:   - 2-chamber - 4-chamber - 3-chamber - Short axis stack | - TR: 9.0ms - TE: 1.2ms - FOV 360 x 360 mm - Slice Thickness: 8mm |
| Late Gadolinium Enhancement | Segmented k-space late gadolinium enhancement acquisitions in multiple planes with Phase Sensitive Inversion Recovery sequence (PSIR) and magnitude reconstructions (MAGIR). | Standard cardiac planes:   - 2-chamber - 4-chamber - 3-chamber - Short axis stack   Further sequence to determine the optimal inversion time. | - TR: 9.8ms - TE: 4.6m - α: 21o - FOV 340 x 220 mm (transverse plane) - Slice thickness 8mm - sampled matrix size 256 x 115-135, 21 k–space lines acquired every other RR interval (21 segments with linear reordered phase encoding) - spatial resolution 1.3 x 2.1 x 8 mm. |

## Table 3. Rapid non-contrast Cardiovascular Magnetic Resonance (CMR) – 6 months and 12 months – 1.5 Tesla (T)

| Cardiovascular Magnetic Resonance (CMR)Technique | CMR Sequence | Scan Prescription | Typical Scan Parameters |
| --- | --- | --- | --- |
| Localizer and Pilots | - Bright blood single shotfast spin-echo stack. - Steadystate free precession (SSFP) | - 3 orthogonal slices (axial, coronal, sagittal) - Transverse stack covering the heart   Standard cardiac planes:   - 2-chamber - Short axis stack (5 slices) | - Repeat time (TR): 2.37ms - Echo time (TE): 1.2ms - Slice thickness 8mm - Field of view (FOV): 360 x 360mm - Red matrix 256 - Flip angle 60. |
| Cine Imaging | - SSFP | Standard cardiac planes:   - 2-chamber - 4-chamber - 3-chamber - Short axis stack | - Retrospective ECG gating - TR: 2.7ms - TE: 1.1ms, - Flip angle 80° - Grappa factor 2 - voxel size 1.8x1.8mmx6mm - x-y spatial resolution1 to 2mm/pixel - slice thickness 8mm.   For SAX:   - inter-slice gap 2mm - 10 segments (true temporal resolution 27ms, reconstructed temporal resolution 22-33) |
| Tissue Mapping | - multiparametric SAturation‐recovery single‐SHot Acquisition (mSASHA) | - mSASHA: 4Ch, SAX (3 slices) | - TR: 9.0ms - TE: 1.3ms - FOV 360 x 360 mm - Slice Thickness: 8mm |

## Supplemental Materials 1 – Participant Informed Consent Form

**IRAS Number:** 312432

**Patient Identification Number:**

**Study Number:**

**INFORMED CONSENT FORM**

**Title of Project:** **Randomised Control Trial for the Safety of Withdrawal of Pharmacological Treatment for Recovered HER2 Targeted Therapy Related Cardiac Dysfunction**

**Sponsor:** University College London

**Funder:** British Heart Foundation

**Chief Investigator:** Professor Charlotte Manisty

Barts Heart Centre, St Bartholomew’s Hospital, EC1A 7BE

**Name of Researchers:** Professor Charlotte Manisty, Professor Malcolm Walker, Dr Alex Lyon, Dr Carla Plymen, Dr Benjamin Dowsing.

**Please initial box**

1. I confirm that I have read and understood the information sheet (Version 3.0) for the above study. I have had the opportunity to consider the information, ask questions and have had these answered satisfactorily.
2. I understand my participation is voluntary and that I am free to withdraw at any time, for any reason, and without my medical care or legal rights being affected.
3. I understand that relevant sections of my medical notes and data collected during the study may be looked at by members of the research team, individuals from University College London, from regulatory authorities or from the Barts Health NHS Trust or University College London Hospital Trust where it is relevant to my taking part in this research. I give permission for these individuals to have access to my records.
4. I agree to my General Practitioner being informed of my participation in the study. I agree to my General Practitioner being involved in the study, including any necessary exchange of information about me between my GP and the research team.
5. I understand why blood samples are being taken, how the samples will be collected, that giving samples for this research is voluntary and give consent for my samples to be used in this study.
6. I understand that electronic health records held by the local NHS Trust and other central UK NHS bodies may be accessed by members of the research team and linked together to provide information about my long-term health status relevant to the purpose and questions of this study. I agree to be contacted by the research team to gather information related to my health relevant to the study.

YES

NO

1. I am happy to receive an anonymised summary report of this study if one is

generated (optional).

YES

No

1. I additionally give consent for my blood samples to be stored in a human tissue authority (HTA) licensed research bank at the trial site and to be used in future ethically approved studies without the need to contact me again (optional).

YES

NO

1. I additionally give consent for DNA analysis (for genes known to be involved in heart muscle problems only) to be conducted on my blood samples in the event of a relapse in my heart function (optional).

YES

NO

1. I additionally give consent for my anonymised data to be shared with collaborators, including potential overseas collaborators, in future ethically approved studies without the need to contact me again (optional).

YES

NO

1. I additionally give consent for the research team to collect data related to my heart

health and cardio-oncology follow up for the 5-year period after my final study visit

(optional).

YES

NO

1. If my stored samples or anonymous data are used in any future research, I would like to receive an anonymised summary report of the study if one is generated (optional).

YES

NO

1. I agree to take part in the above study.

Date

Signature

Name of participant

Name of person taking consent

Date

Signature

1x original – into Site File; 1x copy – to Participant; 1x copy – into medical record.

## Supplemental Materials 2 – Medication Disutility Questionnaire

**1. Generic Medication Usefulness**

Please imagine a tablet which:

- Has no side effects

- Costs you next to nothing to obtain

- You do not need a prescription for it

- There is no problem if you stop it at any time or if you stop and start – except that you might not get the full benefit

If this tablet enabled your heart to stay healthy for an extra ***1* day** of life, do you think you would take it? **YES NO**

*Consider first “1 day” and then “10 years”. If the answers are “no” and “yes” respectively for those first two dates, consider the date at the midpoint (on this chart) between the longest date to which “no” and the shortest date to which the “yes” are the answer. Based on this stepwise process please circle the shortest amount of ‘healthy heart’ time that you would find acceptable for you to take the tablet.*

***1 day***

*1 week*

*2 weeks*

*1month*

*2 months*

*3 months*

*4 months*

*6 months*

***9 months***

*10 months*

*1 year*

*2 years*

*3 years*

*4 years*

*5 years*

*8 years*

***10 years***

2. **The effects of** **your heart medication**

Regarding the heart medications you were prescribed.

Have you experienced any of the following side effects and if yes how often has this occurred?

| **Side Effect** | **Yes/No** | **Daily** | **Weekly** | **Monthly** | **Less than Monthly** |
| --- | --- | --- | --- | --- | --- |
| **Altered Sleep** |  |  |  |  |  |
| **Cough** |  |  |  |  |  |
| **Dizziness** |  |  |  |  |  |
| **Fatigue** |  |  |  |  |  |
| **Low Mood** |  |  |  |  |  |
| **Nausea** |  |  |  |  |  |
| **Nightmares** |  |  |  |  |  |
| **Poor concentration** |  |  |  |  |  |
| **Other (Please enter below)** | | | | | |
|  |  |  |  |  |  |
|  |  |  |  |  |  |

If yes to any of the above, how would you rate how this affected your quality of life?

| **1**  **Not at all** | **2** | **3** | **4** | **5** | **6** | **7** | **8** | **9** | **10**  **Extremely Severely** |
| --- | --- | --- | --- | --- | --- | --- | --- | --- | --- |
|  |  |  |  |  |  |  |  |  |  |

Please explain:

How many other regular medications do you take daily?

| **0** | **1** | **2** | **3** | **4** | **5** | **6** | **7** | **8** | **9** | **10** |
| --- | --- | --- | --- | --- | --- | --- | --- | --- | --- | --- |
|  |  |  |  |  |  |  |  |  |  |  |

How often have you forgotten to take your heart medication or missed a dose?

| **Daily** | **Weekly** | **Monthly** | **Less than Monthly** | **Never** |
| --- | --- | --- | --- | --- |
|  |  |  |  |  |

Do you find taking the heart medications inconvenient? (Consider having to take the medication, collecting repeat prescriptions etc).

| **1**  **Not at all inconvenient** | **2** | **3** | **4** | **5** | **6** | **7** | **8** | **9** | **10**  **Extremely inconvenient** |
| --- | --- | --- | --- | --- | --- | --- | --- | --- | --- |
|  |  |  |  |  |  |  |  |  |  |

Please explain:

Does taking the heart medication have any personal financial cost? (Consider prescription fees, loss of work etc)

| **1**  **No Cost** | **2** | **3** | **4** | **5** | **6** | **7** | **8** | **9** | **10**  **Extremely significant Cost** |
| --- | --- | --- | --- | --- | --- | --- | --- | --- | --- |
|  |  |  |  |  |  |  |  |  |  |

Please explain:

Did taking your heart medications have any effect on family planning? **YES / NO**

Please explain (if you are happy to do so):

**3. The effects on your heart from breast cancer treatment**

Regarding the effects on your heart from your breast cancer treatment.

Do you currently consider yourself to have a heart condition? **YES NO**

Please explain:

How severe do you feel your heart condition was when it was first noticed?

| **1**  **Not at all** | **2** | **3** | **4** | **5** | **6** | **7** | **8** | **9** | **10**  **Extremely Severe** |
| --- | --- | --- | --- | --- | --- | --- | --- | --- | --- |
|  |  |  |  |  |  |  |  |  |  |

Please explain:

How severe do you feel your heart condition currently is?

| **1**  **Not at all** | **2** | **3** | **4** | **5** | **6** | **7** | **8** | **9** | **10**  **Extremely Severe** |
| --- | --- | --- | --- | --- | --- | --- | --- | --- | --- |
|  |  |  |  |  |  |  |  |  |  |

Please explain:

How significantly has your heart condition affected you?

| **1**  **No affect** | **2** | **3** | **4** | **5** | **6** | **7** | **8** | **9** | **10**  **Extremely significant affect** |
| --- | --- | --- | --- | --- | --- | --- | --- | --- | --- |
|  |  |  |  |  |  |  |  |  |  |

Please explain:

How important do you feel the heart medications are in managing this heart condition?

| **1**  **Not at all Important** | **2** | **3** | **4** | **5** | **6** | **7** | **8** | **9** | **10**  **Extremely Important** |
| --- | --- | --- | --- | --- | --- | --- | --- | --- | --- |
|  |  |  |  |  |  |  |  |  |  |

Please explain:

How concerned are you about the potential downsides of stopping the heart medication?

| **1**  **Not concerned at all** | **2** | **3** | **4** | **5** | **6** | **7** | **8** | **9** | **10**  **Extremely Concerned** |
| --- | --- | --- | --- | --- | --- | --- | --- | --- | --- |
|  |  |  |  |  |  |  |  |  |  |

Please explain:

Have you considered stopping the heart medications prior to your involvement in this study?

| **1**  **Never** | **2** | **3** | **4** | **5** | **6** | **7** | **8** | **9** | **10**  **Extremely Frequently** |
| --- | --- | --- | --- | --- | --- | --- | --- | --- | --- |
|  |  |  |  |  |  |  |  |  |  |

Please explain:

**4. Heart Medication Usefulness**

Please imagine the heart medications you were prescribed.

If taking those tablets enabled your heart to stay healthy for an extra ***1 day*** of life, do you think you would have continued to take them? **YES NO**

*As for question 1 - Consider first “1 day” and then “10 years”. If the answers are “no” and “yes” respectively for those first two dates, consider the date at the midpoint (on this chart) between the longest date to which “no” and the shortest date to which the “yes” are the answer. Based on this stepwise process please circle the shortest amount of ‘healthy heart’ time that you would find acceptable for you to continue to take the heart medications you were prescribed.*

***1 day***

*1 week*

*2 weeks*

*1month*

*2 months*

*3 months*

*4 months*

*6 months*

***9 months***

*10 months*

*1 year*

*2 years*

*3 years*

*4 years*

*5 years*

*8 years*

***10 years***
