# Supplementary material for: HER-SAFE study design: an open-label, randomised controlled trial to investigate the safety of withdrawal of pharmacological treatment for recovered HER2-targeted therapy-related cardiac dysfunction
Source: BMJ Open. 2025 Feb 5;15(2):e091917. doi: 10.1136/bmjopen-2024-091917 (PMC11800297; doi:10.1136/bmjopen-2024-091917)
Supplement: Supplementary file 2 [file bmjopen-2024-091917.reviewer_comments.pdf]

# PEER REVIEW HISTORY

BMJ Open publishes all reviews undertaken for accepted manuscripts. Reviewers are asked to complete a checklist review form and are provided with free text boxes to elaborate on their assessment. These free text comments are reproduced below.

## ARTICLE DETAILS

### Title (Provisional)

HER-SAFE study design: An open label, randomised controlled trial to investigate the safety of withdrawal of pharmacological treatment for recovered HER2 targeted therapy related cardiac dysfunction.

### Authors

Dowsing, Benjamin; Dehbi, Hakim-Moulay; Chung, Robin; Pedra, Joanna; Worn, Orla; Artico, Jessica; Schmid, Peter; Roylance, Rebecca; Kellman, Peter; Moon, James C; Crake, Tom; Westwood, Mark; Ghosh, Arjun; Andres, Maria Sol; Nazir, Muhummad Sohaib; Lyon, Alexander R; Chen, Daniel; Walker, Malcolm; Manisty, Charlotte H

---

This article was not externally reviewed at BMJ Open. Protocols that have been independently assessed prior to submission to BMJ Open are usually fast-tracked to publication on the grounds that further substantial changes will not be possible. This independent assessment will usually be external, independent review for both a substantial grant award from a non-commercial or government funder and ethics approval.
